# Supplementary material for: Characterization of the Common Japonica-Originated Genomic Regions in the High-Yielding Varieties Developed from Inter-Subspecific Crosses in Temperate Rice (Oryza sativa L.)
Source: Genes (Basel). 2020 May 18;11(5):562. doi: 10.3390/genes11050562 (PMC7290844; doi:10.3390/genes11050562)
Supplement: Supplementary file 1 [file genes-11-00562-s001.zip › Figure S1.pdf]

Figure S1. Pedigrees of eight HYVs used in this study. (**A**) Tongil, (**B**) Minghui 63, (**C**) Cheongcheongbyeo, (**D**) Milyang 23, (**E**) Nampungbyeo, (**F**) Hanareumbyeo, (**G**) Dasanbyeo, (**H**) Takanari.

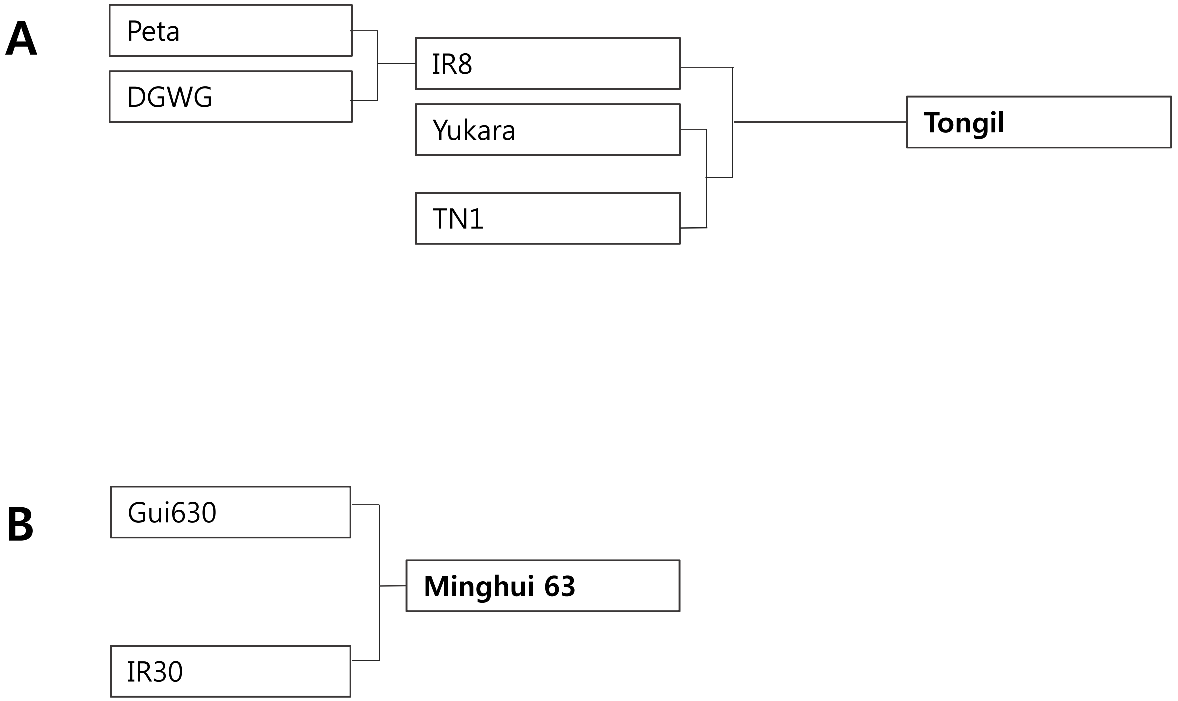

C

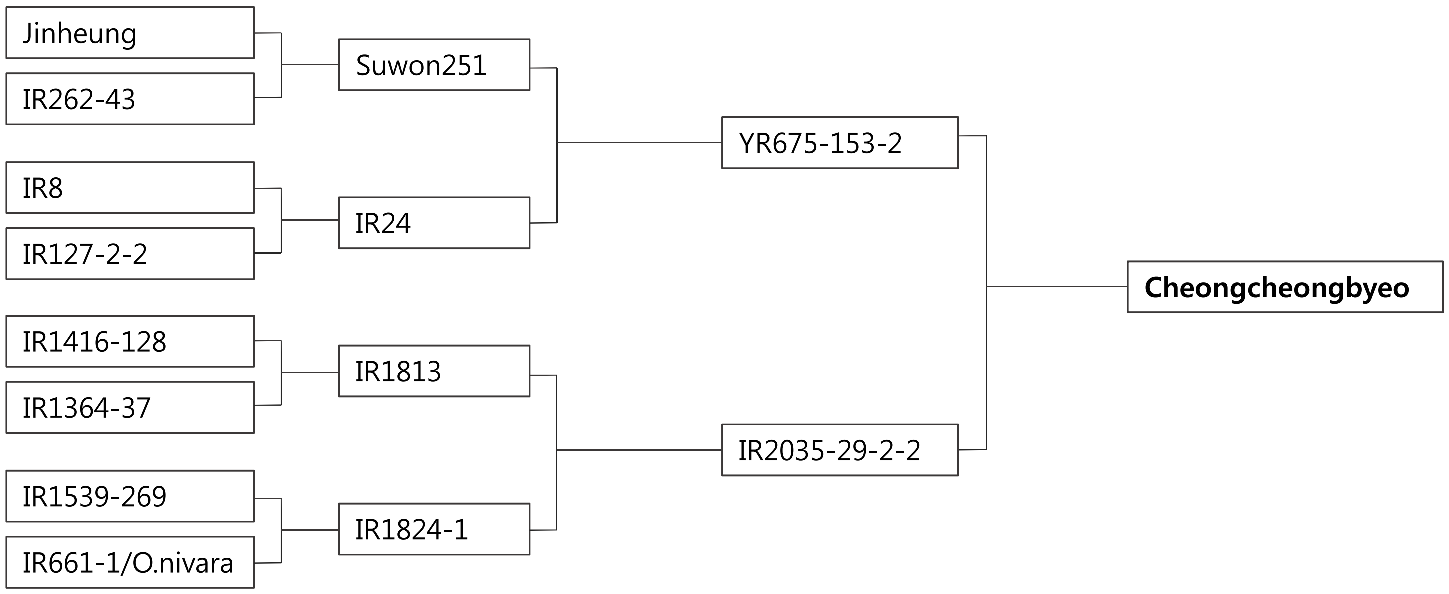
Jinheung
IR262-43
IR8
IR127-2-2
IR1416-128
IR1364-37
IR1539-269
IR661-1/O.nivara
Suwon251
IR24
IR1813
IR1824-1
YR675-153-2
IR2035-29-2-2
Cheongcheongbyeo

D

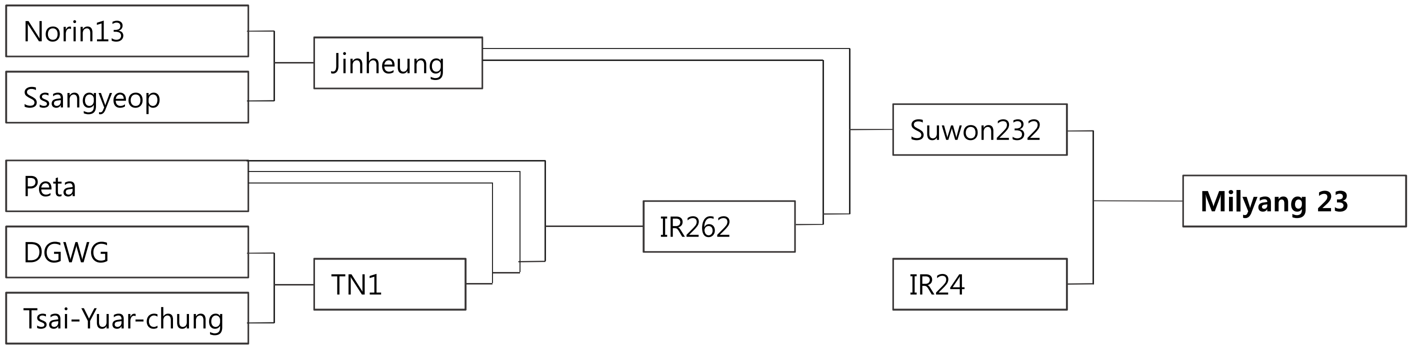
Norin13
Ssangyeop
Peta
DGWG
Tsai-Yuar-chung
Jinheung
TN1
IR262
Suwon232
IR24
Milyang 23

E

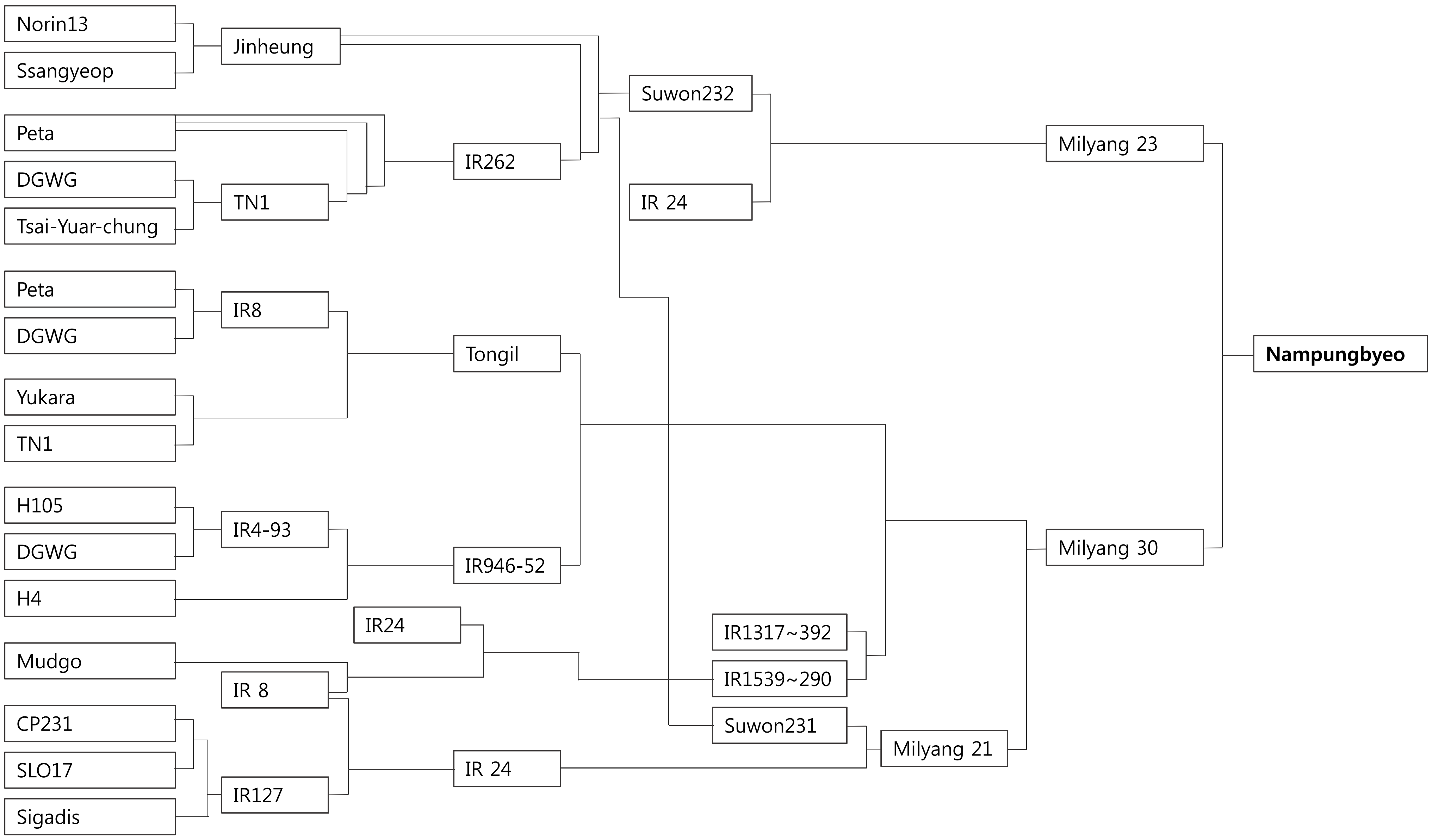
Norin13
Ssangyeop
Jinheung
Peta
DGWG
Tsai-Yuar-chung
TN1
IR262
Suwon232
IR 24
Milyang 23
Peta
DGWG
IR8
Yukara
TN1
Tongil
Nampungbyeo
H105
DGWG
IR4-93
H4
IR946-52
Milyang 30
IR24
Mudgo
IR 8
IR1317~392
IR1539~290
CP231
SLO17
Sigadis
IR127
IR 24
Suwon231
Milyang 21

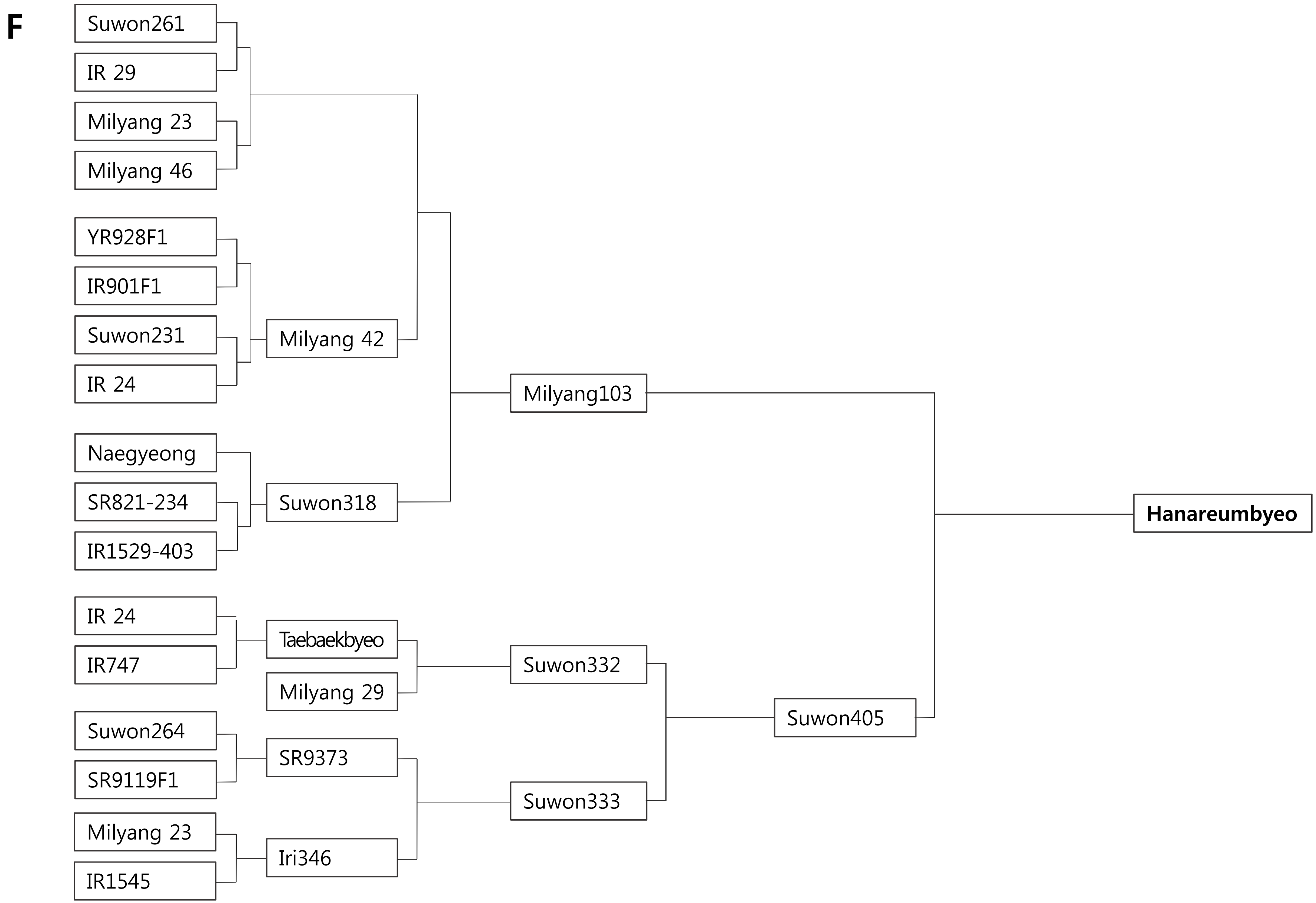
F
Suwon261
IR 29
Milyang 23
Milyang 46
YR928F1
IR901F1
Suwon231
IR 24
Milyang 42
Naegyeong
SR821-234
IR1529-403
Suwon318
Milyang103
IR 24
IR747
Taebaekbyeo
Milyang 29
Suwon332
Suwon264
SR9119F1
SR9373
Milyang 23
IR1545
Iri346
Suwon333
Suwon405
Hanareumbyeo

G

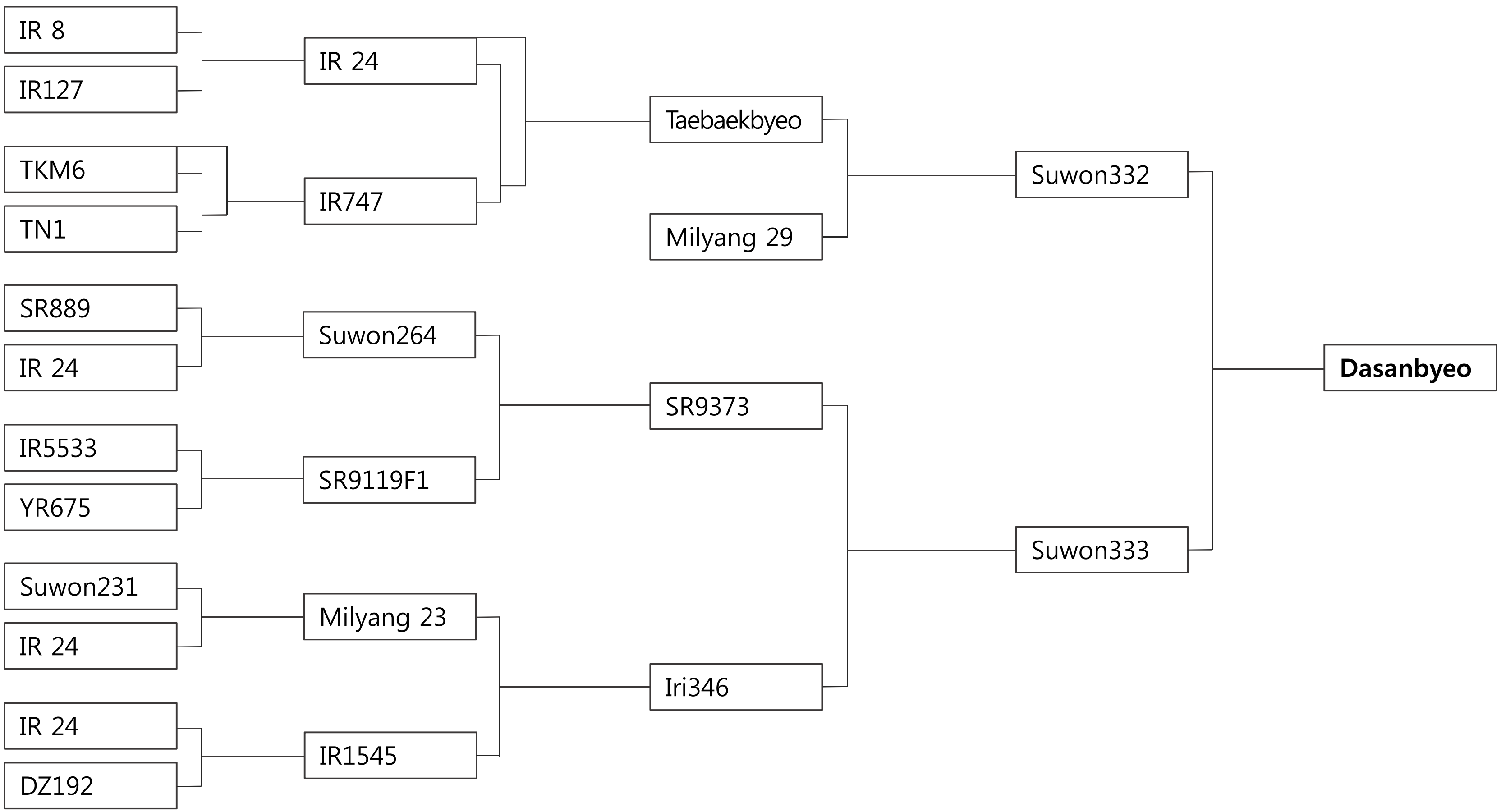

IR 8
IR127
TKM6
TN1
SR889
IR 24
IR5533
YR675
Suwon231
IR 24
IR 24
DZ192
IR 24
IR747
Suwon264
SR9119F1
Milyang 23
IR1545
Taebaekbyeo
Milyang 29
SR9373
Iri346
Suwon332
Suwon333
Dasanbyeo

H

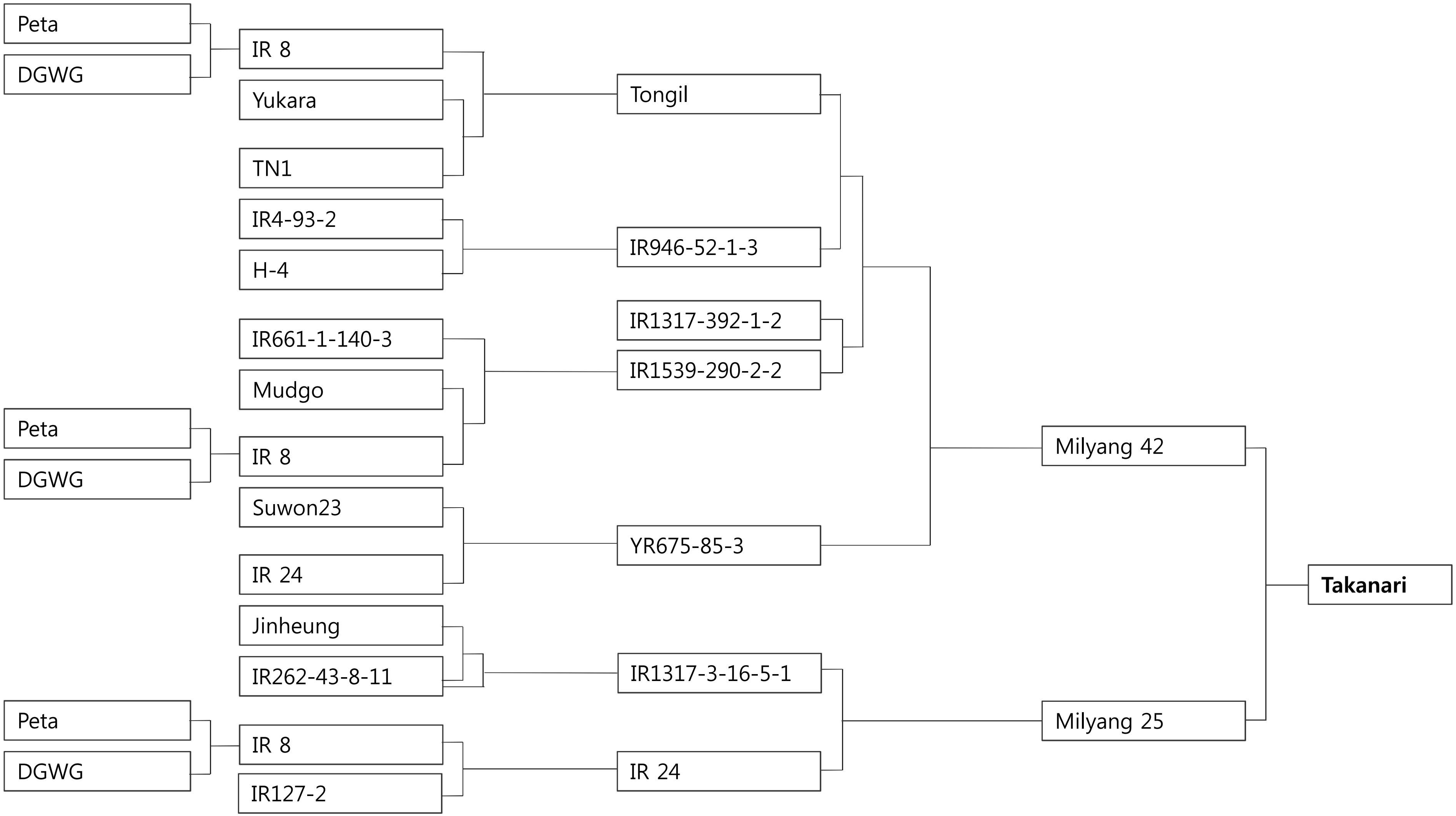
Peta
DGWG
IR 8
Yukara
TN1
IR4-93-2
H-4
Tongil
IR946-52-1-3
IR1317-392-1-2
IR1539-290-2-2
IR661-1-140-3
Mudgo
Peta
DGWG
IR 8
Suwon23
IR 24
YR675-85-3
Milyang 42
Takanari
Jinheung
IR262-43-8-11
IR1317-3-16-5-1
Peta
DGWG
IR 8
IR127-2
IR 24
Milyang 25
